# Rare presentation of rickettsial infection as purpura fulminans: a case report

**DOI:** 10.1186/s13256-018-1672-5

**Published:** 2018-05-26

**Authors:** Chamara Dalugama, Indika Bandara Gawarammana

**Affiliations:** Department of Medicine, University of Peradeniya, Peradeniya, Sri Lanka

**Keywords:** Purpura fulminans, Rickettsiae, Disseminated intravascular coagulation

## Abstract

**Background:**

Purpura fulminans is an acute life-threatening disorder characterized by intravascular thrombosis and hemorrhagic infarction of the skin complicated with disseminated intravascular coagulation. It is commonly seen in acute infections following meningococcal and streptococcal infections. Few cases of purpura fulminans following rickettsial infections have been described in the literature.

**Case presentation:**

We report a case of a 55-year-old Sri Lankan woman who presented to Teaching Hospital Peradeniya with a febrile illness, headache, and myalgia that progressed to an erythematous rash starting over the bilateral lover limbs and hands and that became black and necrotic with a few hemorrhagic blebs. She had normocytic anemia, platelet clumps, and monocytosis as well as a deranged clotting profile. The result of immunofluorescence antibody testing for rickettsial immunoglobulin G was strongly positive for *Rickettsia conorii* with a rise in titer convalescent sera, and a diagnosis of purpura fulminans following rickettsial infection was made. The patient made an excellent recovery with chloramphenicol treatment.

**Conclusions:**

The treating physician should consider the rare but very treatable condition of rickettsial infection as a differential diagnosis in the etiological diagnostic workup of patients presenting with severe purpuric and hemorrhagic rash with fever.

## Background

Purpura fulminans is a rare syndrome of intravascular thrombosis and hemorrhagic infarction of the skin that is rapidly progressive and accompanied by vascular collapse and disseminated intravascular coagulation [1]. Dermal vascular thrombosis can be devastating and associated with significant morbidity and mortality [2]. Three categories are identified: inherited abnormalities of the coagulation system, acute infectious type, and idiopathic type [2]. The commonest group is the acute infections type, most notably with meningococcal, staphylococcal, and streptococcal infection [3–5]. Purpura fulminans caused by rickettsial infection is very rarely reported in the literature. If the etiology of purpura fulminans is promptly diagnosed as a rickettsial infection and treated accordingly, the outcome will be excellent. We report a case of a 55-year-old woman with rickettsial infection who presented with purpura fulminans and was successfully treated with chloramphenicol.

## Case presentation

**Fig. 1:**
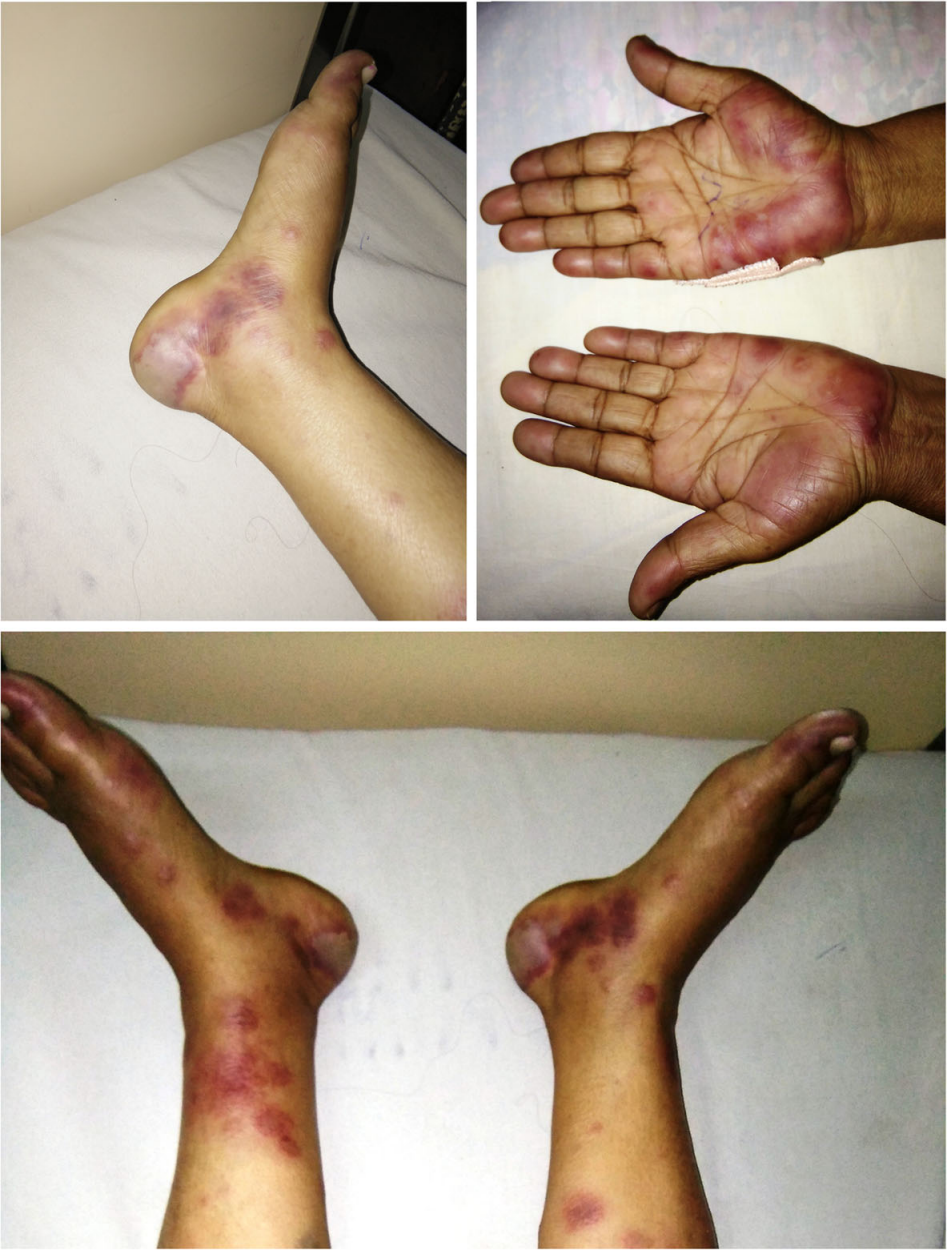
The extensive purpuric rash with areas of hemorrhage and necrosis involving the both legs and the hands

A previously well 55-year-old Sri Lankan Sinhalese woman from Kandy presented to Teaching Hospital Peradeniya with a history of high-grade fever of 7 days’ duration with severe headache. She had anorexia and generalized malaise. She was treated with a course of amoxicillin and clavulanic acid by a general practitioner for possible sinusitis, but she had no clinical response. On day 5 of her febrile illness, she noticed a rash that was erythematosus initially, starting over her bilateral lower limbs and hands and progressing rapidly to involve her back and trunk. The lesions progressed to become black and necrotic with a few hemorrhagic blebs. She had severe pain in her hands and feet, particularly worsening with cold exposure. She had been to southeastern Sri Lanka on a pilgrimage and recalled having multiple tick bites. On examination, she was in severe distress with a high fever of 105 °F. Her higher mental functions were intact. She had marked conjunctival injection. An extensive purpuric rash with areas of hemorrhage and necrosis was seen over both legs, hands, and the trunk (Fig. 1). The results of the rest of the systemic examination were normal. The patient did not have an eschar. She did not have any other bleeding manifestations.

The patient’s complete blood count showed a total white blood cell (WBC) count of 6.6 × 10^9^/L (60% neutrophils, 15% lymphocytes, and 25% monocytes), hemoglobin of 9.1 g/dl, and a platelet count of 36 × 10^9^/L. A peripheral smear taken upon admission showed normochromic normocytic anemia with marked monocytosis and apparently low platelets that could have been due to multiple platelet clumps, and there was no evidence of microangiopathic hemolytic anemia (MAHA), but subsequent peripheral smears drawn on the third and fifth days of admission showed evidence of MAHA with marked thrombocytopenia. The patient’s prothrombin time was 15.7 s (control 12 s), and her activated partial thromboplastin time was 36 s (control 25 s). Her d-dimer level was 1500 ng/ml (normal range < 500 ng/ml). Her fibrinogen levels were not available in the hospital. Her transaminases were within the normal ranges. Her serum protein concentration was 61 g/L with albumin of 30 g/L. The results of her renal function tests were normal. Her serum lactate dehydrogenase was 810 U/L (normal range 225–450 U/L). The results of her blood and urine cultures were sterile. A transthoracic echocardiogram showed normal cardiac valves and endocardium. The result of immunofluorescence antibody testing of rickettsial immunoglobulin G was strongly positive for *Rickettsia conorii*, with a rise in titer convalescent sera repeated after 2 weeks.

Our patient was treated initially with doxycycline. She continued to spike high fevers while on doxycycline, and her skin lesion was spreading extensively. On the third day of her hospital stay, intravenous chloramphenicol was started, and she was afebrile within 24 h. Intravenous chloramphenicol was continued for a total of 7 days. Antipyretics, antiallergy drugs, and topical emollients were also applied. She recovered completely, including regression of the rash at follow-up after 10 days. Her complete blood count showed a WBC of 8 × 10^9^/L with a normal differential count, hemoglobin of 10.5 g/dl, and a platelet count of 156 × 10^9^/L, and a peripheral smear showed complete resolution of features of disseminated intravascular coagulation.

## Discussion

Rickettsiae are a group of alphaproteobacteria found as an obligatory intracellular parasite of eukaryotic cells [6]. Rickettsia is a reemerging infection in Sri Lanka, and three types of rickettsial infections are widely reported: the spotted fever group, murine typhus, and scrub typhus [7, 8]. The clinical presentation of rickettsial infection has a wide spectrum ranging from undifferentiated fever to multiorgan involvement leading to fatal outcomes [9]. Rickettsial infections can present with various cutaneous manifestations. The typical features of the skin rash include discrete macular-papular lesions with a dusky erythematosus hue, distributed mainly in the limbs, the back of the chest, the anterior abdomen, and the soles of the feet [10]. However, there are many other variations, including fern leaf pattern skin necrosis, patchy necrotic lesions, and cutaneous edema [10]. Identification of cutaneous lesions in rickettsial infections plays a pivotal role in making a diagnosis early.

Purpura fulminans is a life-threatening disorder characterized by sudden progressive cutaneous hemorrhage and necrosis. It can be due to inherited defects of hemostatic mechanism such as protein C or S deficiency [11]. Acquired causes of purpura fulminans include infections by *Neisseria meningitidis*, *Streptococcus pneumoniae*, *Haemophilus influenzae*, *Haemophilus aegyptius*, *Staphylococcus aureus*, group A and other beta-hemolytic streptococci, *Pseudomonas aeruginosa*, and *Candida albicans* [12]. The pathophysiology of purpura fulminans is complicated and not yet fully described. Postinfectious purpura fulminans may be caused by an acquired deficiency of protein S. A consistent feature of this condition is development of autoantibodies against protein S [13]. Histopathological hallmarks of acute infectious purpura fulminans are dermal vascular thrombosis and secondary hemorrhagic necrosis, findings that are identical to those of the Shwartzman reaction, which involves a disturbance in the balance of anticoagulant and procoagulant activities of endothelial cells [14].

In the literature, rickettsial infections are very rarely reported to cause purpura fulminans. The evidence comes from a few reported cases. Biradar *et al.* reported a case of a 60-year-old man who presented with purpuric lesions over both upper and lower limbs and consumption coagulopathy following rickettsial infection. He was treated with replacement of platelets and coagulation factors along with antibiotics and doxycycline, and he made an uneventful recovery [15]. Katoch *et al.* described a case series of purpura fulminans that included four pediatric patients treated with doxycycline with good recovery [16]. The few similar cases successfully treated with doxycycline have been reported mainly from the Indian subcontinent [17–19].

The first drug of choice for rickettsial disease is doxycycline. Alternatively, chloramphenicol can be used. Some strains of rickettsiae are poorly responsive to standard antirickettsial drugs, including tetracycline or doxycycline [20]. Some suggest using alternative therapy such as doxycycline or combination therapy including rifampicin in managing resistant cases [21]. Our patient did not have a good response to doxycycline, but adding chloramphenicol led to defervescence within 24 h and dramatic clinical improvement.

## Conclusions

Rickettsia is a reemerging infection in Sri Lanka. Clinical features can range from undifferentiated fever to fatal multiorgan involvement. Cutaneous manifestations are pivotal to early diagnosis of the condition. Rarely, purpura fulminans can be the presentation of rickettsial infection. The treating physician should consider the possibility of rickettsial infection in the etiological diagnostic workup of any patient who presents with a severe purpuric and hemorrhagic rash.
